# Detection and monitoring of human bocavirus 1 infection by a new rapid antigen test

**DOI:** 10.1016/j.nmni.2016.01.015

**Published:** 2016-02-09

**Authors:** A.H.L. Bruning, P. Susi, H. Toivola, A. Christensen, M. Söderlund-Venermo, K. Hedman, H. Aatola, A. Zvirbliene, J.O. Koskinen

**Affiliations:** 1) Department of Pediatric Infectious Diseases, Emma Children's Hospital, Academic Medical Center, Amsterdam, The Netherlands; 2) Department of Virology, University of Turku and Biomaterials and Diagnostics Group, Turku University of Applied Sciences, Turku, Finland; 3) ArcDia International Oy Ltd., Turku, Finland; 4) Trondheim University Hospital and Norwegian University of Science and Technology, Trondheim, Norway; 5) Department of Virology, University of Helsinki, and Helsinki University Hospital, Helsinki, Finland; 6) Department of Immunology and Cell Biology, Institute of Biotechnology, Vilnius University, Lithuania

**Keywords:** Human bocavirus, human bocavirus 1, point-of-care test, rapid antigen detection assay, respiratory infection

## Abstract

Clinically relevant diagnosis of human bocavirus 1 (HBoV1) is challenging, as the virus is frequently detected in asymptomatic patients, and cofindings with other respiratory viruses are common. The clinical value of current diagnostic methods, such as PCR, is therefore low, and alternative diagnostic strategies are needed. We describe for the first time the use of an antigen detection assay for the rapid identification of HBoV1 in a paediatric patient with respiratory tract infection symptoms. We estimate the duration of active HBoV1 infection to be 6 days.

## Introduction

Human bocaviruses (HBoVs) are small single-stranded DNA viruses which belong to the family *Parvoviridae* (subfamily *Parvovirinae*). Currently four HBoV species have been identified. Human bocavirus 1 (HBoV1) was described for the first time in 2005 in children with respiratory tract infection (RTI) [1]. Three other HBoVs, designated HBoV2, HBoV3 and HBoV4, have mainly been detected in faeces, but a causal relation with disease such as gastroenteritis is still unclear [2]. Prevalence studies using nucleic acid amplification methods show that HBoV1 can be detected in 1.6 to 21.5% of children with symptoms of RTI, particularly during winter and spring [3]. Seroepidemiology studies in the United States [4] and Italy [5] indicated that over 90% of children had antibodies for HBoV by 4 years of age.

PCR amplification of viral nucleic acids is the most commonly used technique for the detection of HBoV1 in respiratory samples. However, the true clinical impact of a positive PCR result is often difficult to assess as HBoV1 DNA may persist for months in the respiratory tract. The DNA can be encountered in both symptomatic patients and asymptomatic individuals, as well as a cofinding in respiratory infections caused by other pathogens [6].

Nevertheless, causative associations between HBoV1 infection and severe illness, including unexplained severe lower RTI [7] and encephalitis [8], have been described. This emphasizes the need for novel diagnostic methods for accurate and rapid identification of clinically relevant HBoV1 infections. Several techniques for the diagnosis of HBoV1 infection have been developed. These include serology [9], mRNA reverse transcriptase (RT) PCR [10] and DNA PCR in serum and in nasal samples [11]. However, these methods are cost and labour intensive and are only available in highly specialized diagnostic laboratories.

The mariPOC test system (ArcDia International Oy Ltd., Turku, Finland) provides a rapid alternative for HBoV1 detection. mariPOC is an automated and point-of-care compatible test for rapid and simultaneous detection of antigens of eight respiratory viruses (influenza A and B, respiratory syncytial virus, adenovirus, human metapneumovirus and parainfluenza type 1, 2 and 3 viruses) and *Streptococcus pneumoniae* from a single nasopharyngeal sample. Detection of antigens is based on separation-free two-photon excitation fluorometry [12]. Recently a method to detect HBoV1 antigen was added to the test panel. The new HBoV1 antigen test has an analytical sensitivity of 3 ng/mL for recombinant HBoV-like particles (VP2), and it has shown good correlation with HBoV1 mRNA PCR designed to detect acute infection cases only (Toivola *et al.*, poster presented at 25th European Congress of Clinical Microbiology and Infectious Diseases, 2015).

Here we describe the use of the HBoV1 antigen test for the detection and monitoring of HBoV1 in nasopharyngeal samples.

## Case Report

**Fig. 1:**
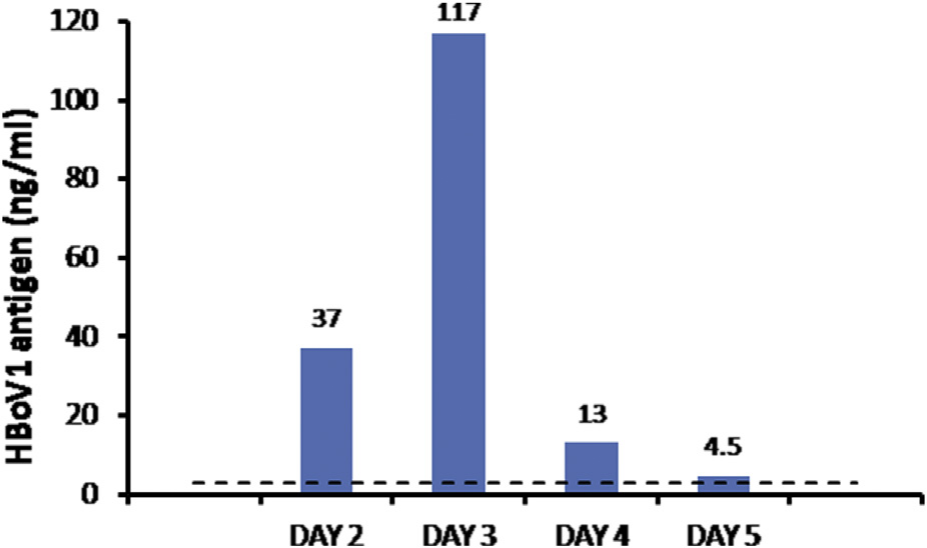
Detection of human bocavirus 1 (HBoV1) antigen by mariPOC test (ArcDia International Oy Ltd., Turku, Finland) during course of respiratory infection. Dashed line indicates cutoff for positive finding. For each day of infection, nasopharyngeal sample was analysed by HBoV1 antigen test.

A previously healthy girl, 5 months of age, developed symptoms of rhinorrhoea, cough and fever (temperature up to 39°C). The consulting paediatrician diagnosed an upper RTI with no lower respiratory tract involvement or signs of otitis. After written parental consent was obtained, a nasopharyngeal swab was taken on day 2 of illness and tested by the mariPOC for virologic diagnosis. Within 20 minutes the sample tested positive for HBoV1 antigen. Retrospective PCR testing confirmed the diagnosis: the nasopharyngeal sample tested positive for HBoV1 DNA by quantitative PCR [13] with a load of 7.7 × 10^7^ copies/mL and for HBoV1 mRNA by RT-PCR [10] with a Ct value of 26. To follow the course of infection, nasopharyngeal samples were taken on days 3, 4 and 5 from the onset of symptoms. While rhinorrhoea and mild cough continued, the fever declined on day 3. An initial increase in mariPOC assay signal was followed by a gradual decrease (Fig. 1). The viral antigen concentrations were extrapolated from a dose–response curve of a positive control dilution series. On day 6, the RTI symptoms were mild, which coincided with a decreasing HBoV1 antigen load.

## Discussion

With the mariPOC rapid test system, we were able to *in vitro* diagnose and monitor for the first time the course of a primary HBoV1 RTI by directly targeting the virus proteins. Compared to standard laboratory diagnostics, this rapid test is easy to perform and may allow for a clinically accurate diagnosis of HBoV1 infection. The initial HBoV1 antigen detection was confirmed by both DNA and mRNA PCR. The costs of the mariPOC multianalyte tests are comparable with other antigen detection assays, such as direct fluorescent antibody testing. Compared to rapid and easy to perform nucleic acid detection methods, e.g. PCR assays with integrated sample preparation, mariPOC immunoassay is a less expensive alternative.

mariPOC test results are typically reported as qualitative. In this study we followed the course of the HBoV1 infection by using the quantitative property of the underlying two-photon excitation technology. However, the nature of swab sampling limits the quantitativeness of the data to semiquantitative. We showed the duration of this HBoV1 active infection to be approximately 1 week. The patient was HBoV1 antigen positive the morning after onset of symptoms, and HBoV1 antigen positivity lasted until day 5, when the signal barely exceeded the diagnostic threshold. No sample was available beyond day 5, while the decline in antigen since day 3 and the near-cutoff result on day 5 strongly suggest that the patient would have been negative on day 6. These results are in line with the time span of other respiratory viruses [14] and demonstrate once more that rapid testing should be done as soon as possible after the onset of symptoms and at the latest within 5 to 6 days.

While most HBoV1 infections are self-limited, they sometimes lead to life-threatening conditions. The availability of a rapid and accurate test for HBoV1 is likely to be useful in differential diagnoses and development of therapy, and will increase our understanding of the epidemiology and clinical impact of HBoV1 infections.

## References

[1] Allander T., Tammi M.T., Eriksson M., Bjerkner A., Tiveljung-Lindell A., Andersson B. (2005). Cloning of a human parvovirus by molecular screening of respiratory tract samples. Proc Natl Acad Sci U S A.

[2] Paloniemi M., Lappalainen S., Salminen M., Katka M., Kantola K., Hedman L. (2014). Human bocaviruses are commonly found in stools of hospitalized children without causal association to acute gastroenteritis. Eur J Pediatr.

[3] Dunn J.J., Miller M.B. (2014). Emerging respiratory viruses other than influenza. Clin Lab Med.

[4] Kahn J.S., Kesebir D., Cotmore S.F., D'Abramo A., Cosby C., Weibel C. (2008). Seroepidemiology of human bocavirus defined using recombinant virus-like particles. J Infect Dis.

[5] Guido M., Zizza A., Bredl S., Lindner J., De Donno A., Quattrocchi M. (2012). Seroepidemiology of human bocavirus in Apulia, Italy. Clin Microbiol Infect.

[6] Martin E.T., Kuypers J., McRoberts J.P., Englund J.A., Zerr D.M. (2015). Human bocavirus 1 primary infection and shedding in infants. J Infect Dis..

[7] Moesker F.M., van Kampen J.J., van der Eijk A.A., van Rossum A.M., de Hoog M., Schutten M. (2015). Human bocavirus infection as a cause of severe acute respiratory tract infection in children. Clin Microbiol Infect.

[8] Mitui M.T., Tabib S.M., Matsumoto T., Khanam W., Ahmed S., Mori D. (2012). Detection of human bocavirus in the cerebrospinal fluid of children with encephalitis. Clin Infect Dis.

[9] Kantola K., Hedman L., Allander T., Jartti T., Lehtinen P., Ruuskanen O. (2008). Serodiagnosis of human bocavirus infection. Clin Infect Dis.

[10] Christensen A., Dollner H., Skanke L.H., Krokstad S., Moe N., Nordbo S.A. (2013). Detection of spliced mRNA from human bocavirus 1 in clinical samples from children with respiratory tract infections. Emerg Infect Dis.

[11] Allander T., Jartti T., Gupta S., Niesters H.G., Lehtinen P., Osterback R. (2007). Human bocavirus and acute wheezing in children. Clin Infect Dis.

[12] Koskinen J.O., Vainionpaa R., Meltola N.J., Soukka J., Hanninen P.E., Soini A.E. (2007). Rapid method for detection of influenza A and B virus antigens by use of a two-photon excitation assay technique and dry-chemistry reagents. J Clin Microbiol.

[13] Kantola K., Sadeghi M., Antikainen J., Kirveskari J., Delwart E., Hedman K. (2010). Real-time quantitative PCR detection of four human bocaviruses. J Clin Microbiol.

[14] Suryaprasad A., Morgan O.W., Peebles P., Warner A., Kerin T.K., Esona M.D. (2011). Virus detection and duration of illness among patients with 2009 pandemic influenza A (H1N1) virus infection in Texas. Clin Infect Dis.

